# Risk of Porcine reproductive and respiratory syndrome virus introduction into Danish pig farms: a register-based study

**DOI:** 10.1186/s40813-026-00489-2

**Published:** 2026-02-06

**Authors:** Mette Fertner, Søren Kjærgaard Boldsen, Nicolai Rosager Weber, Janni Hales Pedersen, Nils Toft

**Affiliations:** 1 Livestock Innovation, SEGES Innovation P/S, Agro Food Park 15, Aarhus N, 8200 Denmark; 2 Danish Agriculture & Food Council, Copenhagen, Denmark

**Keywords:** PRRS, Swine, Pigs, Disease transmission, Movement data, Risk factors

## Abstract

**Background:**

Porcine reproductive and respiratory syndrome (PRRS) remains one of the most important virus infections in the industrialised pig production sector. In 2022, a national PRRS control programme was launched in Denmark. Nevertheless, new cases of PRRS-positive farms continue to be reported. The objective of the present study was to identify routes of introduction and risk factors associated with the introduction of the PRRS virus onto Danish pig farms.

**Results:**

Based on register data from 2023, we identified 175 cases (173 farms) that changed their serological status from PRRS-negative to PRRS-positive, and 1,909 controls (1,665 farms) that remained PRRS-negative. The overall incidence was estimated to be 9.5% (175/1,838), with an incidence of 5.7% (39/788) for farms with sows and 11.8% (136/1,151) for weaner and/or finisher farms. Inward movement of PRRS-positive pigs was identified as the most likely route of introduction for 20% (8/40) of the farms with sows and 82% (112/136) of the weaner and/or finisher farms. Cases resulting from the deliberate inward movement of known PRRS-positive pigs were excluded from the dataset before logistic regression models were developed with case/control as a response variable predicting the risk of PRRS introduction. Two logistic regression models were developed: one for farms with sows and one for weaner and/or finisher farms. Evaluated risk factors included farm size, number of supplying farms, the supplier becoming PRRS-positive within 90 days of movement, distance to PRRS-positive neighbours and season. The two final multivariable models included both two highly significant risk factors: the supplying farm becoming PRRS-positive within 90 days of movement of pigs (p < 0.001) and the distance to PRRS-positive neighbours (p < 0.001). Crude observations identified a 16 and 43 times higher risk of becoming PRRS-positive if the supplier became PRRS-positive within 90 days of movement of pigs, compared with farms where the supplier remained PRRS-negative (RRsow = 15.7 [9.1;27.3] CI95%, RR_WF_ = 42.5 [28.3;64.0] CI95%). Likewise, there was a four and nine times higher risk of farms becoming PRRS-positive if PRRS-positive neighbours were located within a 5 km radius, compared with no positive neighbours within this distance (RRsow = 4.2 [1.8;10.6] CI95%; RR_WF_ = 8.5 [3.4;20.9] CI95%). On-farm biosecurity measures that have been identified as major factors in other studies were not possible to include, as this study was based purely on register-data.

**Conclusions:**

The results from the present study confirm that the movement of pigs is the main driver of PRRS infections on Danish weaner and/or finisher farms. Local transmission from neighbouring farms appears to be of secondary importance.

## Background

Porcine reproductive and respiratory syndrome (PRRS) caused by the PRRS virus (PRRSV) remains one of the most economically important diseases in the industrialised pig production sector [1].

PRRSV can spread between farms by the direct movement of infected pigs, by indirect contacts, air and semen. The movement of pigs is considered to be the most common route of PRRS transmission between farms, especially for weaner farms [1, 2]. Furthermore, indirect contacts such as personal, contaminated equipment [3], trucks or other vehicles have also been associated with PRRS transmission [1, 2, 4], while airborne spread of PRRSV has been found at distances of 5.8 km [5] and 9.1 km [1]. However, transmission by air is not fully understood. The potential of airborne spread appears to be dependent on the PRRSV type and weather conditions [1]. Conditions favouring airborne viral spread include low velocity wind, low temperature, high relative humidity and low ultraviolet light (sunlight) [1].

Routes of infection differ between farm types. While movements were identified as the main driver of disease transmission for weaner farms, accounting for 80% of the infections, local transmission dominated infections on sow farms, accounting for 59% of the infections [2].

Previous risk factor studies have found that the size of the farm, on-farm biosecurity measures and the PRRSV status of neighbours impact the risk of PRRSV infection. Increased farm size has been found to be positively associated with the risk of PRRSV infection [6] as well as an increased risk of being seropositive for PRRSV [7]. Likewise, the introduction of large numbers of animals [8] and the proximity to infected farms [1] have been found to increase the risk of PRRSV introduction. Furthermore, on-farm biosecurity measures, such as the isolation of purchased gilts, decreased the risk of PRRSV infection [9], while compromised gilt quarantine [7] has been found to impact the risk of PRRSV infection significantly.

In 2022, a national control programme for PRRS was launched in Denmark [10]. As part of the programme, all farms with more than ten sows or 100 pigs in total are obliged to have a serological PRRS health declaration status assigned. The PRRS health declaration of a farm can have the status of *negative*, *positive*, *under eradication* or *unknown*. A PRRS health declaration status is based on annual serological testing from 20 individual pigs, while the inward movement of pigs from a PRRS-positive supplying farm will automatically result in a PRRS-positive status [11]. Breeding/multiplier farms have monthly serological antibody testing performed, due to their voluntary enrolment in the industry-driven Specific Pathogen Free programme [12]. Hence, 4.6% of the farms (breeding/multiplier) are tested monthly, while 95.6% of the pig (production) farms are tested yearly for antibodies in serum [13]. In addition to this, PRRS is a notifiable disease, which means that the farmer and veterinarian are obliged to report clinical symptoms and submit sufficient material for PCR analysis. In the following text, PRRS-negative and PRRS-positive refer to farms with a negative and positive PRRS health declaration status, respectively.

Several registers available in Denmark contain information on the majority of pig farms, and they facilitate population-based studies of a number of factors. The Central Husbandry Register (CHR) includes information on farm location, farm size, animal species, ownership and movement of animals. The CHR is a public register owned by the Danish Veterinary and Food Administration in which all pigs are obliged to be registered [14]. The Specific Pathogen Free (SPF) register is an industry-driven register. By January 2024, 52% (2,288/4,420) of all industrialised Danish pig farms had an SPF health declaration status, while 95% (4,211/4,420) had a PRRS health status [15]. For a farm to enrol in the SPF health declaration system, it needs to fulfil certain biosecurity standards in terms of, for example, entry of visitors and pigs as well as the testing frequency of the seven pathogens included in the system. Hence, the SPF register contains information on the level of biosecurity and health status regarding the seven pathogens included in the SPF system (SPF-Sund 2024). Enrolment in the SPF system is typically driven by an economic incentive, since the declared SPF health status typically affects the price of the weaners sold. The PRRS health declaration status is registered in the SPF register, where non-SPF farms with a PRRS status appear as PRRS-declared farms.

Despite the national control programme, new cases of PRRS-positive farms continue to be reported in Denmark. Therefore, there is a need to identify both routes of introduction and factors that impact the risk of a farm becoming PRRS-positive.

The objective of the present study was to identify routes of introduction and risk factors associated with the introduction of the PRRS virus onto Danish pig farms by using register data.

## Materials and methods

### Study design

We performed a retrospective longitudinal study based on data available in the national registers. The model was developed based on data from 1 January to 31 December 2023, while the model validation was performed based on data from 1 January to 30 August 2024.

### Data

Data from the following two databases were used: the SPF database (historical information on declared PRRS status) [12] and the Central Husbandry Register (geographical farm location, farm size and registered movements of pigs) [14]. All data extractions were performed on 18 November 2024.

### Study population

All production farms meeting the definition of either being a Case or a Control were included in the study. Newly infected farms (Cases) were identified in the register data as farms that had experienced a change in the declared PRRS status from negative to positive at a given date. For comparison, non-infected farms (Controls) were defined as farms that were most likely assumed to be PRRS-negative. Since a PRRS-negative status can be maintained for up to one year without further sampling, there is a risk of the farm becoming infected during this time period. The longer the time since testing, the greater the risk of a false negative status. Therefore, controls were defined as farms that, at a given date, had a negative laboratory ELISA sample submitted and additionally a negative PRRS health status during the entire previous three months. Multiplier/breeding farms were not included in the study since they differ substantially from production farms in their level of biosecurity, incidence rate of PRRS and testing frequency. To align with the aim of identifying risk factors for farms becoming newly infected with PRRS, farms with an unknown PRRS health status or those undergoing eradication were excluded from being classified as either case or control. However, all farms were included as a neighbouring effect in the parameter *distance to PRRS-positive neighbours*.

In addition, all farms were defined as one of three farm types (sow farms / farrow-to-finish farms / farms with weaners and/or finishers). These types of farm were defined based on the number of pigs registered in the CHR. Sow farms were defined as farms with sows registered in numbers that exceeded the number of finishers registered, since farms with finishers registered may apply to the own production of gilts due to the size of the animals falling into the same category registered in the CHR. Farrow-to-finish farms were defined as farms with fewer sows registered than finishers. Weaner and/or finisher farms had no sows registered.

Due to the variation in management practices and previous publications highlighting the variation in routes of infection between farm types, the analyses were performed separately for farms with sows (sow farms and farrow-to-finish farms) and farms with only weaners and/or finishers.

### Routes of introduction and risk factors

For both sow farms and farms with only weaners and/or finishers, we first identified those farms that had deliberately introduced pigs from a PRRS-positive herd. This means that, at the time of movement, the supplying farm was classified as *PRRS-positive*, *under eradication*, or had an *unknown* status. According to the PRRS health declaration, any PRRS-negative farm that receives pigs from a PRRS-positive supplier will automatically be registered as PRRS-positive. Cases of new PRRS-positive farms resulting from the intentional introduction of PRRS-positive pigs were excluded from the risk factor analysis. However, these farms were still included as potential sources of infection for neighbouring farms when assessing local transmission effects. In the remaining dataset, risk factors to be evaluated included farm size, number of supplying farms, distance to PRRS-positive neighbours, season and the supplier becoming PRRS-positive within 90 days of movement. These risk factors were defined as follows:

**Farm size** was based on the number of pigs registered in the CHR and was calculated as Heat-Producing Units (HPU) to obtain a comparable measure across farm types [6]:

$$\eqalign{ HPU & = 0.45*{N_{sows}} + 0.17*{N_{finishers}} \cr & + 0.10*{N_{weaners}} \cr}$$

Due to the skewed distribution of HPU, this parameter was categorised as small farms (20–300 HPU), medium-sized farms (300–600 HPU) or large farms (600-2,380 HPU).

**Number of supplying farms** was based on the registered movements: the number of suppliers within the last 365 days was counted for each farm and categorised as no suppliers, one supplier or two or more suppliers.

**Supplier becoming PRRS-positive within 90 days of movement** (yes/no): If at least one supplying farm changed its PRRS health status to positive within 90 days after moving pigs, this was recorded. In other words, we identified farms where a supplier was declared PRRS-positive within 90 days after selling pigs to the farm of interest. Because serological testing is performed annually and PRRS infection may not always cause detectable clinical signs, newly infected farms may not be identified immediately. Consequently, a supplying farm might sell pigs in good faith, believing them to be PRRS-free.

**Distance to PRRS-positive neighbours** was defined as having PRRS-positive farms within a 1 km radius, within a 5 km radius or no neighbours, meaning that the nearest PRRS-positive farm was located further than 5 km away.

**Season** was defined as each quarter of the year (Jan-Mar, Apr-Jun, Jul-Sep, Oct-Dec) in which the PRRS health declaration status change had occurred (Cases) or in which the negative laboratory sample submission was taken (Controls).

### Statistical analyses

Statistical analyses were performed to quantify the risk of a farm becoming PRRS-positive by means of a logistic regression with case/control as a response variable. Potential risk factors to be evaluated are presented above (farm size, number of supplying farms, risk from neighbouring farms, season and the supplier becoming PRRS-positive within 90 days of movement).

Initially, all risk factors were tested individually in separate univariable analyses. If found to be significant (*p* < 0.05), pairwise post-test comparisons were performed using the emmeans package in R [16]. Afterwards, a multivariable analysis was performed with all risk factors sufficiently associated with the outcome of interest (*p* < 0.20 in the univariable analyses, Table 1). The initial full model was subsequently reduced by stepwise backward elimination until all remaining risk factors were found significant (*p* < 0.05) and subsequently checked for confounding. Odds ratios of the remaining significant risk factors were quantified as an exponentiation of the coefficients and confidence intervals from the final model. Predicted probabilities along with confidence intervals from the final model were estimated based on the predict function in R [17].

Model validation was performed on data from the subsequent year, assuming that the identified effects are constant from one year to the other. Predictions from the model were compared with actual findings on whether the farm became infected. Predicted probabilities were defined as either test positive or test negative and compared with actual PRRS status in the validation dataset. Subsequently, model validation was performed in line with a diagnostic test evaluation. Sensitivity, specificity, positive predicted value and negative predicted values were quantified using the epiR package [18].

To quantify the impact of risk factors of interest, we further estimated the relative risk and population attributable fraction for each significant risk factor and the outcome of interest, assuming a causal relationship and lack of confounding. This was done using the Epitools by AusVet, summarising univariable measures of association from a 2 × 2 table for a cohort study [19].

Data management and statistical analyses were performed in R version 4.3.0 [17], with the use of the packages tidyverse [20] for data management and visualisation, while the lme4 [21] package was used to fit the model.

## Results

### Descriptive statistics

The initial dataset for analysis included 2,158 observations (cases and controls) from 1 January to 31 December 2023. Farms defined as boar stations (*n* = 33) or affiliated quarantine facilities (*n* = 24) were excluded, in addition to farms registered with fewer than ten sows or 100 pigs in total (*n* = 13) and weaner and/or finisher farms without registered inward movements of pigs (*n* = 4). This resulted in a dataset with 2,084 observations (175 cases and 1,909 controls). Controls represented 1,665 farms, with 1,424 farms occurring once, 235 farms occurring twice, and five farms occurring three times in the dataset. Cases represented 173 farms, all occurring once except for two farms, which were represented twice. These two farms had 120 and 215 days registered between reinfection, respectively. The overall incidence of infection^1^ was 9.5% (175/1,838), with an incidence of 5.7% (39/788) for farms with sows and 11.8% (136/1,151) for weaner and/or finisher farms.

Farms with registrations of direct inward movement of pigs from farms with a PRRS-positive or unknown status included 38.3% (67/175) cases and 0.0% (3/1,909) controls. All observations with a deliberate inward movement of PRRS-positive pigs were related to weaner and/or finisher farms. These were deleted prior to the risk factor analyses.

**Fig. 1:**
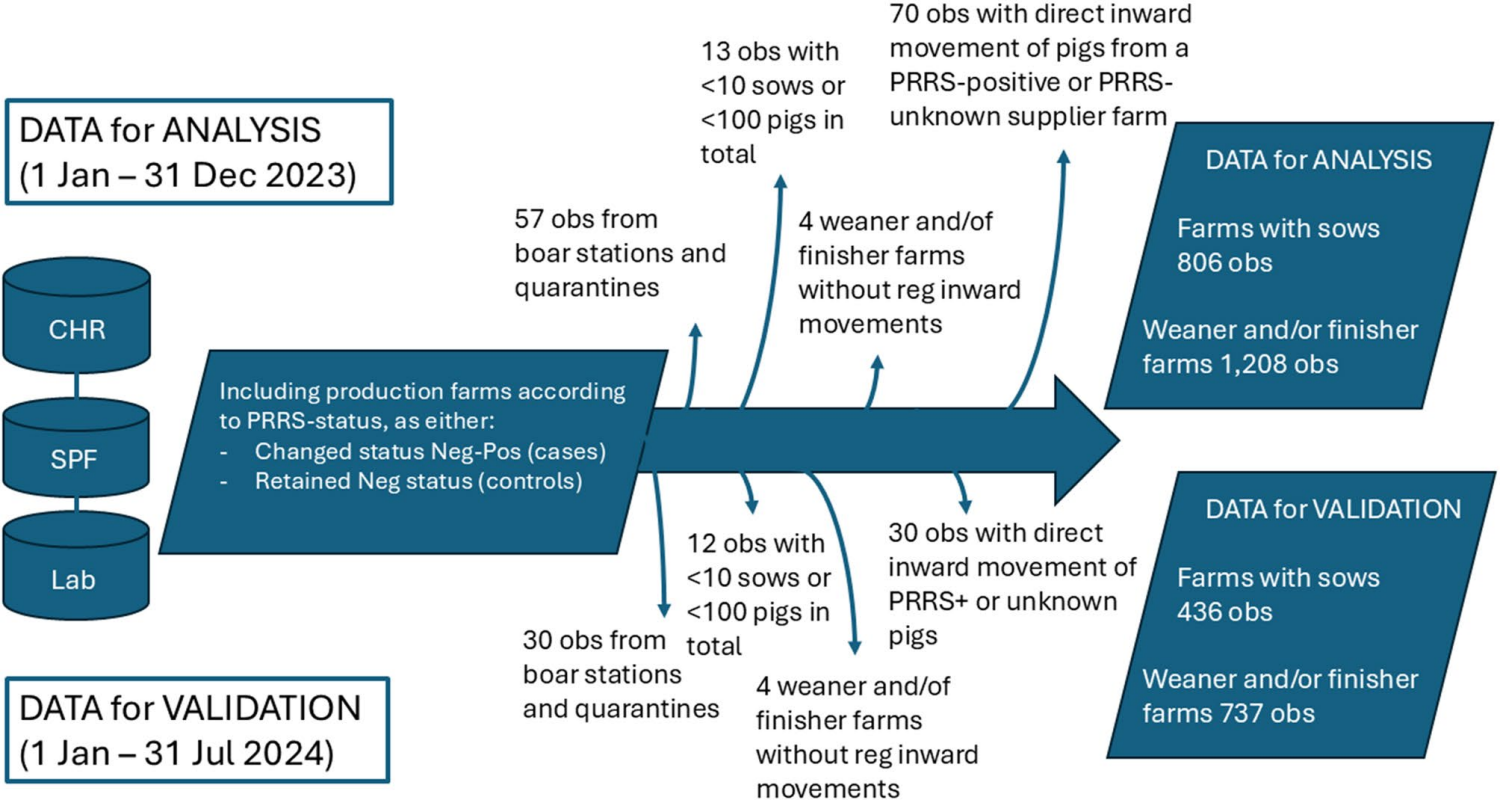
Flowdiagram presenting the data preparation, modelling the risk of farms becoming PRRS-positive. Data were initially extracted from three databases (CHR, SPF and laboratory samples) and separated into one of two study populations (sow farms and weaner and/or finisher farms). The model development was conducted on data from 1 Jan – 31 Dec 2023, while the model validation was performed on data from 1 Jan – 31 Jul 2024

The final two datasets for risk factor analyses included 806 observations (farms with sows) and 1,208 observations (farms with weaners and/or finishers). Among the observations for analysis, 24 farms reoccurred in the dataset as both case and control. Among controls, 234 farms occurred twice, while five farms occurred three times in the dataset. For case farms, only one farm occurred twice (Fig. 1).

In general, a significantly higher proportion of PRRS cases were found on farms with PRRS-positive neighbours located within a 1 km and 5 km radius, while a significantly higher proportion of PRRS-positive cases were found on farms whose supplier became PRRS-positive within 90 days of movement of the pigs.

There was a tendency towards a higher proportion of case farms arising during the winter, with 67% (26/39) and 75% (52/69) of the cases respectively for farms with sows and weaner and/or finisher farms occurring from October to March, compared with 44% (342/767) and 46% (534/1,139) controls in the same period. For the specific periods, a significantly higher proportion of cases on farms with sows was seen from January to March compared with July to September, while none of the other seasons differed from one another. For weaner and/or finisher farms, January to March had a significantly higher proportion of cases compared with any of the other seasons, while October to December also had a significantly higher proportion of cases compared with July to September.

Of the 11 farms without PRRS-positive neighbours in Tables 1; 33% (2/6) of farms with sows and 80% (4/5) weaner and/or finisher farms received pigs from a supplier which became PRRS-positive within 90 days of movement. Hence, 13% (5/39) sow farms and 3% (2/69) weaner and/or finisher farms neither had a positive neighbouring farm nor a supplier becoming infected within 90 days of inward movement of pigs.

**Table 1:** Farm-level risk factors of Danish Sow farms and weaner and/or finisher farms becoming PRRS-positive from 1 January to 31 December 2023

Risk factor		Farms with sows				Weaner and/or finisher farms			
		Cases (*N* = 39)	Controls (*N* = 767)	*P*-value	OR [CI_95%_]	Cases (*N* = 69)	Controls (*N* = 1,139)	*P*-value	OR [CI_95%_]
Number of farms		39	648	-	-	68	1,015	-	-
Farm size (HPU)	20–300	6 (0.046)	125 (0.954)	0.827	1 [1;1]	37 (0.068)	503 (0.931)	**0.165**	1 [1;1]
	300–600	15 (0.044)	326 (0.956)		0.96 [0.38;2.74]	19 (0.041)	439 (0.959)		0.59 [0.33;1.03]
	600–2,380	18 (0.054)	316 (0.946)		1.18 [0.49;3.34]	13 (0.062)	197 (0.938)		0.90 [0.45;1.68]
Number of suppliers within last year	None	6 (0.037)	157 (0.963)	0.219	1 [1;1]	-	-	0.488	-
	1	15 (0.040)	362 (0.960)		1.08 [0.43;3.09]	42 (0.061)	645 (0.939)		1 [1;1]
	2+	18 (0.068)	248 (0.932)		1.90 [0.78;5.33]	27 (0.052)	494 (0.948)		0.84 [0.51;1.37]
Supplier becoming PRRS-positive within 90 days of movement	No	31 (0.039)	762 (0.961)	**< 0.001**	1 [1;1]	24 (0.021)	1,133 (0.979)	**< 0.001**	1 [1; 1]
	Yes	8 (0.615)	5 (0.385)		39.33 [12.42; 136.83]	45 (0.882)	6 (0.118)		354.06 [147.77;999.85]
Distance to PRRS-positive neighbours (within radius)	None	6 (0.017) ^a^	341 (0.983)	**< 0.001**	1 [1;1]	5 (0.010) ^ab^	476 (0.990)	**< 0.001**	1 [1;1]
	5 km	17 (0.041) ^b^	395 (0.959)		2.45 [1.00; 6.84]	37 (0.059) ^ac^	592 (0.941)		5.95 [2.54; 17.40]
	1 km	16 (0.340) ^ab^	31 (0.660)		29.33 [11.22; 86.85]	27 (0.276) ^bc^	71 (0.724)		36.20 [14.63;109.55]
Season	Jan – Mar	17 (0.098) ^a^	156 (0.902)	**0.009**	1 [1;1]	23 (0.139) ^acd^	142 (0.861)	**< 0.001**	1 [1;1]
	Apr – Jun	6 (0.037)	156 (0.963)		0.35 [0.13; 0.87]	9 (0.043) ^a^	200 (0.957)		0.28 [0.12;0.60]
	Jul – Sep	7 (0.025) ^a^	269 (0.975)		0.24 [0.09;0.57]	8 (0.019) ^bc^	405 (0.981)		0.12 [0.05;0.27]
	Oct – Dec	9 (0.046)	186 (0.954)		0.44 [0.19; 1.00]	29 (0.069) ^bd^	392 (0.931)		0.46 [0.26;0.82]

Superscript lower-case letters indicate significance in the probability of PRRS occurrence between strata of the given variable

The results are based on register data. Cases (*N* = 108) define farms that change their status from PRRS-negative to PRRS-positive, while controls (*N* = 1,906) remain negative three months prior to a negative laboratory submission. Farms may reappear in the dataset, which is why the number of observations (2,014) exceeds the number of farms. Farm size is quantified as the number of Heat-Producing units (HPU). P-values are derived from a univariate analysis of a logistic regression between case/control as outcome and each of the risk factors of interest. Risk factors sufficiently associated with the outcome of interest (p-values < 0.2) are highlighted in bold and are further included in one of two multivariable models. Counts (proportion). Odds ratios and associated 95% confidence intervals (OR [CI_95%_]) are presented for univariate analyses

**Table 2:** Movement-related causes of farms becoming PRRS-positive in relation to farm type for 175 new PRRS-positive cases (173 farms) in Denmark in 2023

	Farms with sows^a^	Weaner and/or finisher farms
Deliberate inward movement of PRRS-positive pigs	0	67 (0.493)
Inward movement of potentially infected pigs (supplier becoming PRRS-positive within 90 days of movement)	8 (0.205)	45 (0.331)
Cases not related to movement	31 (0.795)	24 (0.176)
Total	39	136

^a^ The 39 cases included six cases on farrow-to-finish farms and 33 cases on sow farms

Cases in the two categories *inward movement of potentially infected pigs* and *cases not related to movement* (108 cases in total) were included in a subsequent risk factor analysis. Counts (proportion)

Movement-related causes of farms becoming PRRS-positive are presented in Table 2. Deliberate inward movement of PRRS-positive pigs will always result in the receiving farm gaining a PRRS-positive status. These 67 cases were therefore omitted from the dataset prior to the risk factor analysis. Inward movement of potentially infected pigs are cases where the supplier became PRRS-positive within 90 days of movement. If we assume these cases to be due to the movement of false negative PRRS pigs, a total of 69% (120/175) of movement-related cases were found, represented as 21% (8/39) of the farms with sows and 82% (112/136) of the weaner and/or finisher farms. The remaining 31% (55/175) of unknown cases were dominated by farms with sows (Table 2).

For the eight sow farms registered with a supplier becoming positive 90 days after the inward movement had occurred, the owner of the sow farm was also the owner of the supplying farm in all eight cases (Table 2). In most cases, the supplying farm was registered as a weaner and/or finisher farm, which may indicate an inward movement of breeding gilts produced by the farmer himself but bred at another farm. If the farmer does not breed his own gilts in this way, Danish farms typically purchase gilts from breeding/multiplier farms enrolled in the SPF system as Red SPF farms. Such farms have monthly serum samples taken and maintain high external biosecurity standards, which decreases the risk of unknown diseases.

### Results from the risk factor analysis

**Fig. 2:**
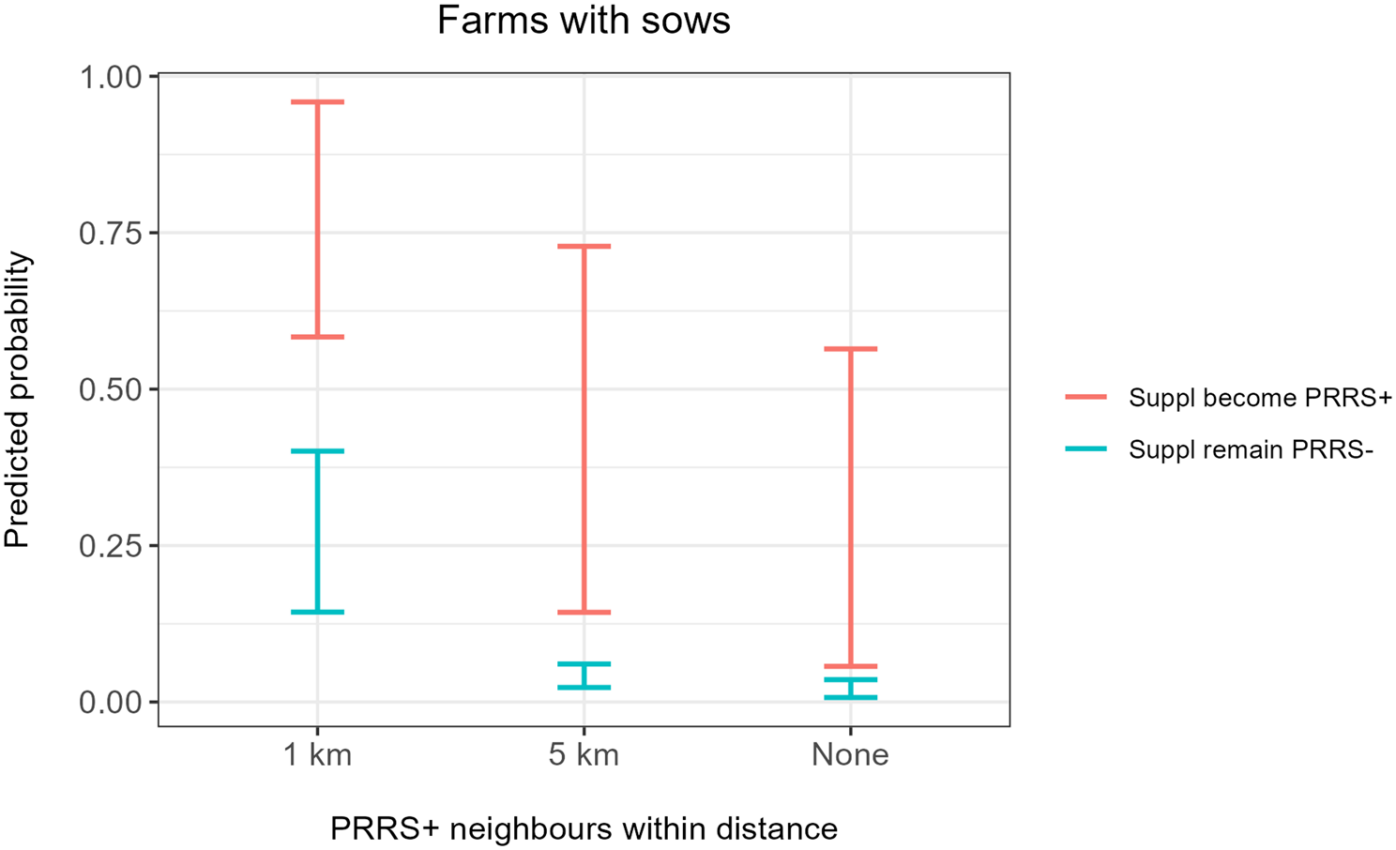
Predicted probabilities from the final multivariable model illustrating risk of farms with sows becoming PRRS-positive. The model includes two significant risk factors, namely distance to PRRS-positive neighbours (x-axis) and whether at least one supplier becomes PRRS-positive within 90 days of movement (colours). Ranges illustrate 95% confidence intervals. The model was based on 806 observations from Danish registers in 2023

**Fig. 3:**
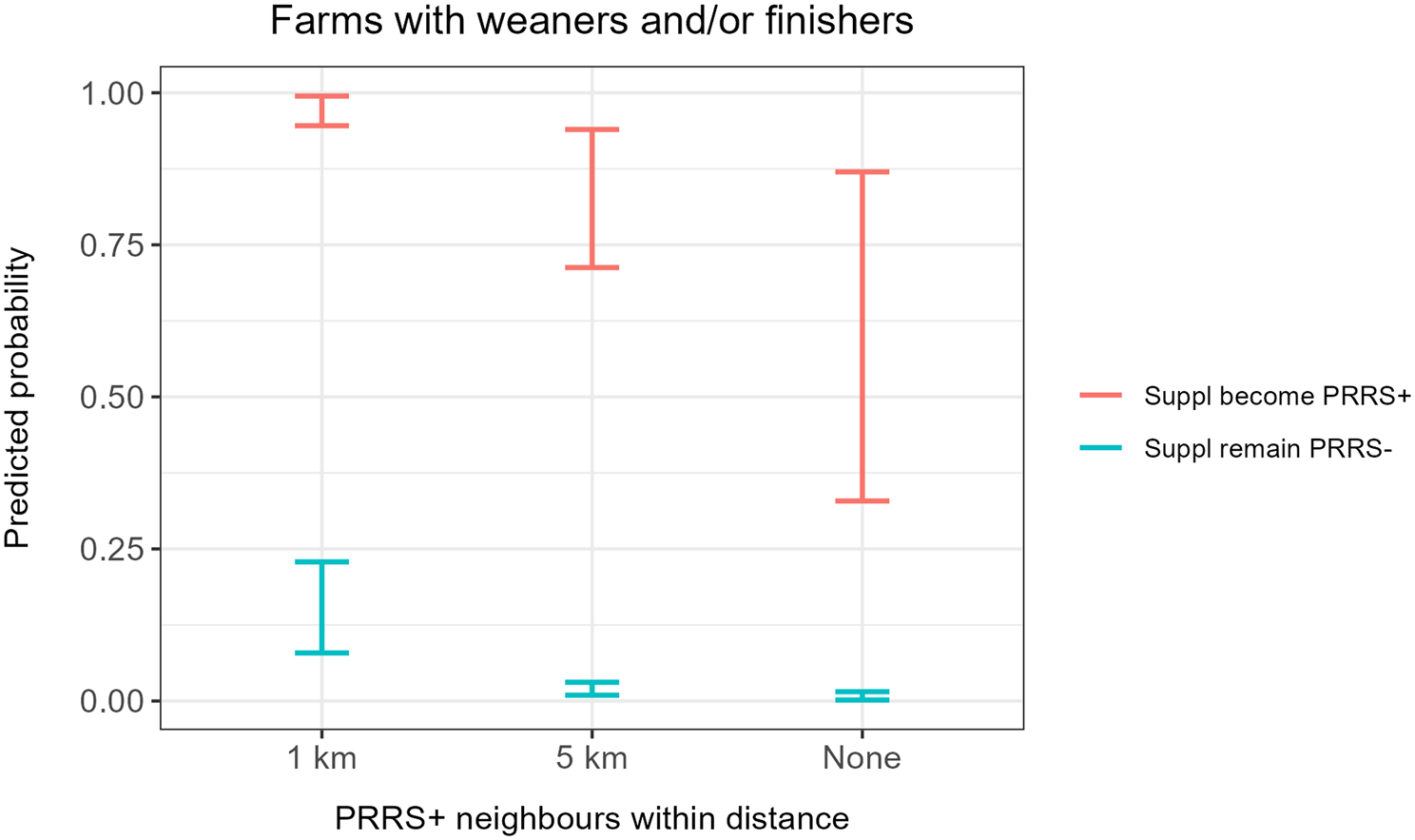
Predicted probabilities from the final multivariable model illustrating risk of weaner and/or finisher farms becoming PRRS-positive. The model includes two significant risk factors, namely distance to PRRS-positive neighbours (x-axis) and whether at least one supplier becomes PRRS-positive within 90 days of movement (colours). Ranges illustrate 95% confidence intervals. The model was based on 1,208 observations from Danish registers in 2023

For farms with sows, the initial univariable screening identified three risk factors of interest to be sufficiently associated with the outcome of interest, namely supplier becoming PRRS-positive, distance to PRRS-positive neighbours and season. For weaner and/or finisher farms, the initial univariable screening identified five risk factors of interest, namely farm size, number of suppliers within the last year, supplier becoming PRRS-positive, distance to PRRS-positive neighbours and season. For both study populations, subsequent stepwise backward elimination revealed two identical highly significant risk factors: distance to PRRS-positive neighbours and supplier becoming PRRS-positive (Tables 3 and 4). Predicted probabilities of both models, for farms with sows and farms with weaners and/or finishers, are illustrated in Figs. 2 and 3.

The relative risk and the population attributable fraction were quantified based on crude observations between new PRRS-positive farms and each of the risk factors in Table 1. We found a 16- and 43-times higher risk of becoming PRRS-positive for farms where the supplier became PRRS-positive within 90 days of movement of the pigs, compared with farms where the supplier remained PRRS-negative (RR_sow_ = 15.7 [9.1;27.3] CI95%, RR_WF_ = 42.5 [28.3;64.0] CI95%). In addition, the population attributable fractions of 19% (farms with sows) and 64% (weaner and/or finisher farms) indicate the proportion of new PRRS-positive cases to be prevented if the suppliers were known to have a true negative status, most likely due to a false negative status of the supplier at the time of delivery of pigs. The three categories were dichotomised in order to quantify these measures of association for distance. The risk of having PRRS-positive neighbours within a 5 km radius was compared with no neighbours. Here, we found a four- and nine-times higher risk of becoming PRRS-positive if the neighbours were located within a 5 km radius compared with no neighbours (RR_sow_ = 4.2 [1.8;10.6] CI95%; RR_WF_ = 8.5 [3.4;20.9] CI95%). Likewise, we would expect 64% (farms with sows) and 82% (weaner and/or finisher farms) of new PRRS-positive cases to be prevented if no farms had PRRS-positive neighbours within a 5 km radius.

**Table 3:** Final model of risk factors impacting the risk of Danish farms with sows becoming PRRS-positive. Data included 806 observations, representing 687 farms with either a change in the PRRS health declaration status from PRRS-negative to PRRS-positive (39 cases) or farms with retained negative status and additional negative laboratory submission samples (767 controls) from 1 January to 31 December 2023

Risk factors		Estimate	SE	OR [CI_95%_]	*p*-value
Intercept		-5.277	0.567	-	
Distance to PRRS-positive neighbours (within radius)	No neighbours	0 (reference)	-	1	< 0.001
	5 km	0.875	0.485	2.39 [0.97; 6.75]	
	1 km	3.018	0.540	20.45 [7.32; 63.00]	
Supplier becoming PRRS-positive within 90 days of movement	No	0 (reference)	-	1	< 0.001
	Yes	2.838	0.704	17.07 [4.33; 70.88]	

**Table 4:** Final model of risk factors impacting the risk of Danish weaner and/or finisher farms becoming PRRS-positive. Data included 1,208 observations, representing 1,083 farms, with either a change in the PRRS health declaration status from PRRS-negative to PRRS-positive (69 cases) or farms with retained negative status and additional negative laboratory submission samples (1,139 controls) from 1 January to 31 December 2023

Risk factors		Estimate	SE	OR [CI_95%_]	*p*-value
Intercept		-4.921	0.459		
Distance to PRRS-positive neighbours (within radius)	No neighbours	0 (reference)	-	1	< 0.001
	5 km	1.23	0.611	3.43 [1.12; 12.74]	
	1 km	3.44	0.646	31.23 [9.59; 125.28]	
Supplier becoming PRRS-positive within 90 days of movement	No	0 (reference)	-	1	< 0.001
	Yes	5.87	0.524	354.49 [136.02;1084.72]	

The effect of distance appears to be similar for both sow farms and weaner and/or finisher farms (Figs. 2 and 3).

### Model validation

Model validation was performed by comparing model predictions with actual observations for the time period 1 January – 31 July 2024. This period included a total of 1,173 observations (63 cases/true positive and 1,110 controls/true negative). Model validation was performed assuming the model was a diagnostic test and assuming predicted probabilities above 0.10 were test positive (9/19 farms with sows; 30/44 weaner and/or finisher farms), while probabilities below 0.10 were assumed to be test negative (399/417 farms with sows; 645/693 weaner and/or finisher farms). The cut-off of 0.10 was based on an estimated overall incidence of PRRS of 9.5%.

**Fig. 4:**
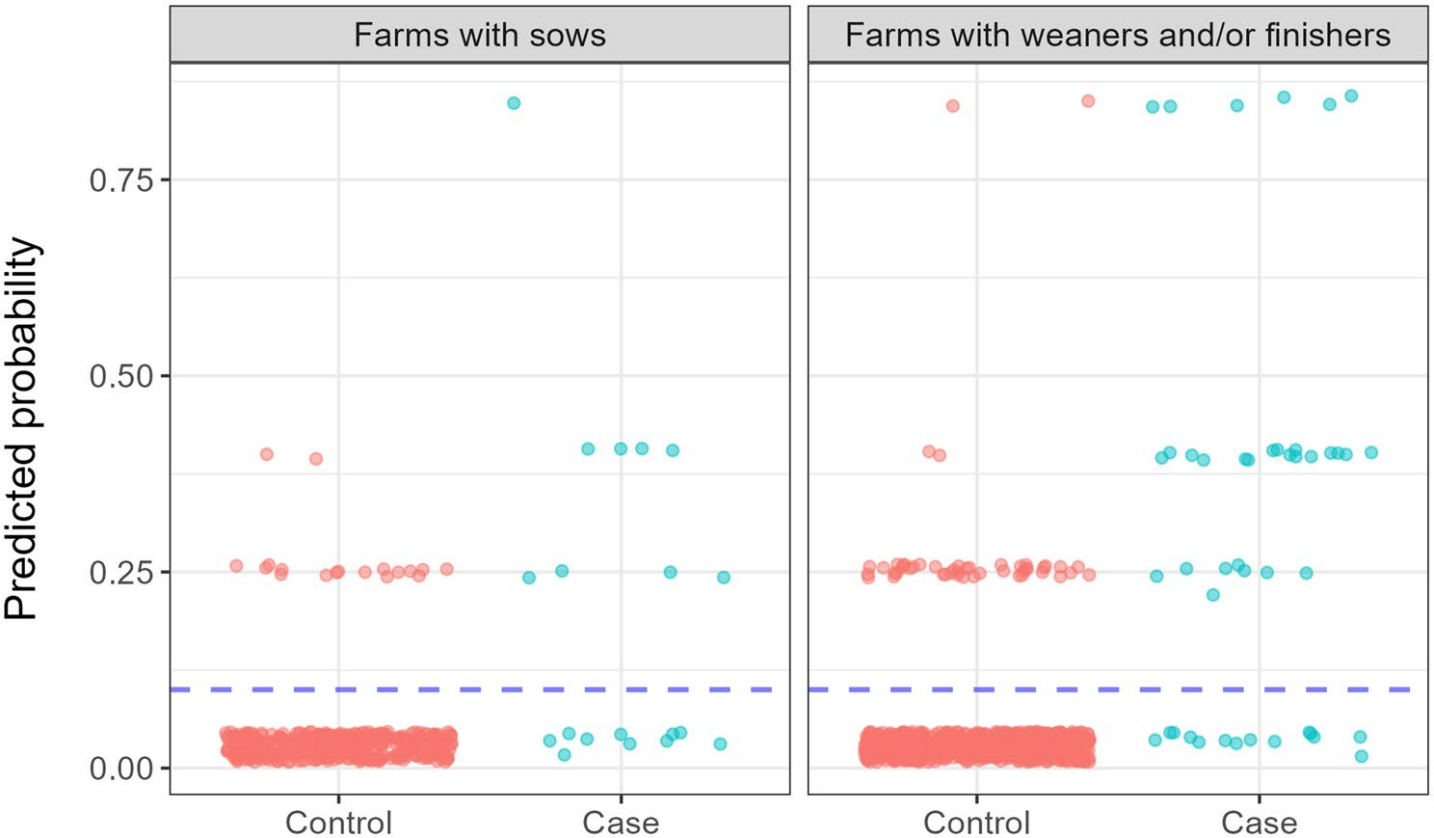
Model validation, illustrating predicted probabilities compared with actual findings in the successive year. Results from two final multivariable models predicting the risk of farms with sows (left) and weaner and/or finisher farms (right) becoming PRRS-positive. The models were based on data from 2023. Predictions are compared with actual findings in 2024 as to whether the farm became PRRS-positive (case) or remained PRRS-negative (control)

Predicted probabilities compared with actual findings are illustrated in Fig. 4. For each model, the sensitivity, specificity, positive predicted value and negative predicted value were calculated (Table 5).

**Table 5:** Model evaluation

Sensitivity	Farms with sows	Weaner and/or finisher farms
	0.47 [0.24;0.71] CI95%	0.68 [0.52;0.81] CI95%
Specificity	0.96 [0.93;0.97] CI95%	0.93 [0.91; 0.95] CI95%
Positive predicted value	0.33 [0.17; 0.54] CI95%	0.38 [0.28; 0.50] CI95%
Negative predicted value	0.98 [0.96; 0.99] CI95%	0.98 [0.96; 0.99] CI95%

Results from two models predicting the risk of PRRS-infection were compared with actual findings of PRRS-infection. Predicting the probability of farms with sows (left) and weaner and/or finisher farms (right) becoming PRRS-positive. Predictions are compared with actual findings as to whether the farm became PRRS-positive or not the successive year. The evaluation is therefore performed in line with a diagnostic test, and the sensitivity, specificity, positive predicted value and negative predicted value are calculated for each of the two models

## Discussion

The results from the present study identify movement of pigs as the main cause of PRRS transmission to Danish weaner and/or finisher farms. Hence, 69% of the new PRRS-positive pig farms were explained by the inward movement of PRRS-infected pigs as the most likely route of introduction. Of movement-related cases, 56% (67/120) were due to deliberate inward movement of PRRS-positive pigs, while 44% (53/120) of the cases were most likely due to inward movement of potentially undetected PRRS-positive pigs. Testing of PRRS antibodies is conducted annually for most Danish pig farms [13]. This relatively rare frequency of testing may increase the risk of moving false negative pigs, meaning pigs which truly harbour the PRRS virus but where the supplying farm is declared negative. However, it is not possible to identify retrospectively in the register data the exact order in which the pig farms became infected. Nevertheless, the purchase of animals from herds incubating PRRS infection has previously been found to pose an increased risk of PRRS transmission [6].

Large discrepancies in routes of introduction were found between farm types in the present study (Table 2). While movements were the most likely cause of introduction for 82% (102/136) of the weaner and/or finisher farms, this only applied to 20% (8/40) of farms with sows (Table 2). This is in line with previous studies which found movements to be the most common route of PRRS transmission between farms, especially nursery farms [1, 2]. The impact of movement may reflect the differences in pigs and procedures of insertion. In Denmark, inward movement of pigs onto sow farms is typically restricted to gilts originating from a multiplier and breeding farm. Multiplier and breeding farms are tested for PRRS antibodies monthly [12], in contrast to the remaining farms, which are tested yearly [10]. Furthermore, gilts are often introduced to the sow farm through a quarantine facility, where it is recommended they stay for 42 days until the results from the antibody testing on the farm of origin are available. In a questionnaire survey of Danish pig producers, 71% of sow farmers reported that they followed the recommendations in terms of strict introduction of gilts for a minimum of 42 days. This indicates that almost 30% of farmers deviate from the recommendations, which may result in the introduction of disease. Nevertheless, the high testing frequency with monthly sampling on multiplier and breeding farms ensures a high probability of the pigs being truly negative for PRRS, and this may compensate for any compromised biosecurity measures.

All pigs on weaner and/or finisher farms originate from a sow farm. The weaner and/or finisher farm may either have a fixed supplier, for example a single sow farm from which they receive all the pigs on a regular basis, or they may have several suppliers. Sow farms are generally tested once a year [10]. Since PRRS may remain undetected due to the lack of clinical signs, the risk of a false negative status increases as time passes since the last testing. Hence, the population attributable fractions of 19% on sow farms and 64% on weaner and/or finisher farms, indicate the percentage of cases which most likely could have been prevented if the suppliers were known to have had a true negative status. Both the population attributable fractions and the relative risks (RR_sow_ = 15.7 [9.1;27.3] CI95%, RR_WF_ = 42.5 [28.3;64.0] CI95%) are crude observations and may overestimate the risk as they do not account for potential confounding factors, such as local transmission and biosecurity measures. Hence, both the population attributable fractions and the relative risks should be interpreted with caution. For the sow farms, this percentage expresses an internal issue for relatively few owners, since all eight cases were caused by the movement of pigs between their own farms. For weaner and/or finisher farms, this percentage expresses a more general issue, which most likely could be prevented if testing was performed more frequently on the supplying farms. This assumes that the owners of the weaner and/or finisher farms do not want to receive PRRS-positive pigs and that they have contractual arrangements that allow them to refuse to receive PRRS-positive pigs. Most farmers purchase pigs from the same supplier every second or third week. When the supplier remains fixed, the health status of the receiving farms is expected to mirror that of the supplier. Therefore, if the supplier acquires a new, undetected PRRS infection, it is highly likely that the receiving farm will also become infected. The testing frequency has previously been a matter of concern for other routes of infection. The introduction of PRRS to a boar station in 2019 resulted in a widespread AI-driven outbreak on Danish sow farms. This resulted in intensified surveillance of boar stations, which are now tested for virus weekly [22]. Hence, the risk of PRRS infection from AI is now considered negligible in Denmark.

When a farm changes its status from PRRS-negative to PRRS-positive, all trading partners will be assigned a derived conditional status, which will be followed up by serological testing in accordance with the SPF regulations. This means that the suppliers of the case group in the current study have a higher chance of testing compared with the suppliers of the control group. The risk of gaining a PRRS-positive status within 90 days of movement of pigs will therefore automatically increase among suppliers of the case group compared with the suppliers of the control group, which may bias the results.

In addition to the inward movement of potentially infected pigs, the results from the risk factor analysis confirmed that distance to PRRS-positive neighbours is a significant risk factor. Likewise, previous simulation studies have estimated that infection on most sow farms is caused by local transmission (e.g. airborne, shared equipment) [2]. In Denmark, local conditions may count for even more due to the widespread use of joint operations managed by the SPF system [12]. Under the SPF system, a group of farms may enrol in a joint operation, so that they will be regarded as one unit of disease status. Hence, all farms will have identical disease status, while employees, equipment and pigs can be moved freely between farms enrolled in the joint operation [12]. Unfortunately, it was not possible to take account of joint operations in the present study due to the lack of historical data on joint operational structures, which may bias the effect of local transmission in the present study. The sharing of equipment and staff between farms under the same ownership may pose an increased risk of disease transmission. A recent study indicated that 2% of farms shared equipment (the washing robots), while 10% of farms shared staff with other farms under the same ownership [23]. One could therefore expect an increased risk of disease transmission between farms under the same ownership, a risk which may have risen in recent years due to the shift in farm demographics to larger farms and a greater number of farms being owned by fewer owners.

Due to the risk of local transmission, it is crucial that PRRS is controlled on a regional level. Relatively soon after the Danish national control programme had been initiated, the country was split into PRRS administrative regions, each with an affiliated coordinator and veterinarian [13]. Recently, the first area, the island of Bornholm, was declared free from PRRS.

The two developed models were evaluated in line with a diagnostic test. In general, the model for weaner and/or finisher farms performed the best in terms of sensitivity, probably because the majority of infections were caused by the movement of pigs and the suppliers changed their status after movement of pigs. There were more unexplained cases on sow farms where the route of infection cannot be found in the register data. Hence, 13% (5/39) of sow farms and 3% (2/69) of weaner and/or finisher farms had neither a positive neighbouring farm nor a supplier that became infected within 90 days of inward pig movements. The source of infection in these cases must therefore be attributed to factors not included in this study.

Although the models were based solely on data from national registers—containing general information on farm type, demographics, and location, but not PRRS-specific details—they performed relatively well. For weaner and/or finisher farms, the model was estimated to detect 68% (95% CI: [0.52; 0.81]) of newly infected PRRS cases. For sow farms, the sensitivity was lower at 47% (95% CI: [0.24; 0.71]), which aligns with the higher proportion of unexplained cases mentioned above.

A PRRS prediction model based on register data, estimating the risk of infection for each individual Danish pig farm, could be a useful tool for authorities in the PRRS reduction program. However, as demonstrated in this analysis, the most accurate models relied on prospective data inputs—such as whether supplying farms became infected within 90 days after pig movements. This limits the predictive value of such models for proactive disease prevention.

Nevertheless, the findings from this study highlight the main transmission pathways of PRRS, which are critical for authorities to consider in disease control efforts. They emphasize the importance of frequent testing of supplier farms to prevent the spread of undetected infections through pig movements. Furthermore, the risk of local transmission underscores the need for regional initiatives as part of a coordinated national strategy to combat PRRS.

## Conclusion

The results of the present study indicate pig movements and local transmission as primary drivers of PRRS virus transmission in Denmark. Movement-related transmission seems to have the highest impact on weaner and finisher farms through the acquisition of pigs from sow farms.

The factors included in this study did not account for all observed cases, which may suggest other undetected routes of transmission to be present, for especially sow farms. These could potentially be linked to biosecurity breaches or contact with external sources not captured in the current dataset.

Results from the present study are critical for efforts aimed at regional control and eventual elimination of PRRS from the Danish pig population. They underscore the importance of targeted interventions, especially in relation to pig movements and enhanced biosecurity protocols.

## Data Availability

The data that support the findings of this study are available from the Danish Veterinary and Food administration (Central Husbandry Register) and Danish Agriculture & Food Council (SPF Register and Laboratory submission data), but restrictions apply to the availability of these data, which were used under license for the current study, and are thereby not publicly available. Data are, however, available from the authors upon reasonable request and with permission from the Danish Veterinary and Food administration and Danish Agriculture & Food Council.

## Footnotes

1 The incidence was estimated roughly as the number of new cases divided by the number of farms at risk at the beginning of the study. Hence, the exact time at risk was not accounted for.
